# CRISPR/Cas12a-based biosensing platform for the on-site detection of single-base mutants in gene-edited rice

**DOI:** 10.3389/fpls.2022.944295

**Published:** 2022-09-07

**Authors:** Mengyu Wang, Xiaojing Liu, Jiangtao Yang, Zhixing Wang, Haoqian Wang, Xujing Wang

**Affiliations:** ^1^Key Laboratory on Safety Assessment (Molecular) of Agri-GMO, Ministry of Agriculture and Rural Affairs, Biotechnology Research Institute, Chinese Academy of Agricultural Sciences, Beijing, China; ^2^Development Center for Science and Technology, Ministry of Agriculture and Rural Affairs, Beijing, China

**Keywords:** single-base mutant detection, fast visual detection, on-site detection, CRISPR/Cas12a, recombinase polymerase amplification

## Abstract

At present, with the accelerated development of the global biotechnology industry, novel transgenic technologies represented by gene editing are developing rapidly. A large number of gene-edited products featuring one or a few base indels have been commercialized. These have led to great challenges in the use of traditional nucleic acid detection technology and in safety regulation for genetically modified organisms (GMOs). In this study, we developed a portable clustered regularly interspaced short palindromic repeats/CRISPR-associated proteins 12a-based (CRISPR/Cas12a-based) biosensing platform named Cas12aFVD (fast visual detection) that can be coupled with recombinase polymerase amplification (RPA) for on-site detection of mutants in gene-edited rice in one tube. The detection procedure can be accomplished in 40 min with a visible result, which can be observed by the naked eye under blue light (470–490 nm). By accurate recognition of targets based on Cas12a/CRISPR RNA (crRNA), Cas12aFVD exhibits excellent performance for the detection of two- and three-base deletions, one-base substitution, and one-base insertion mutants with a limit of detection (LOD) of 12 copies/μl showing great potential for mutant detection, especially single-base mutants. The Cas12aFVD biosensing platform is independent of laboratory conditions, making it a promising and pioneering platform for the detection of gene-edited products.

## Introduction

Three main types of gene-editing tools, zinc finger nucleases (ZFNs) (Pabo et al., 2001), transcription activator-like effector nucleases (TALENs) (Li et al., 2011), clustered regularly interspaced short palindromic repeats/CRISPR-associated proteins (CRISPR/Cas) (Cong et al., 2013), especially CRISPR/Cas, have become the most popular transgenic tools, providing new opportunities in gene function research and breeding (Alberts, 2012). Compared with genetically modified organisms (GMOs) technology, gene editing leaves fewer traces in the body of the target organism. Traditional GMO detection techniques are difficult to meet the requirements for gene-edited products, especially to detect one or a few base changes. While gene editing plays a great role in promoting traditional breeding, it also poses challenges for safety supervision. Therefore, it is necessary to strengthen the research on new detection technologies and establish detection methods for new biotechnological products, such as gene-edited products.

At present, Sanger sequencing (Ma et al., 2015), T7 endonuclease I (T7EI) (Li et al., 2013; Vouillot et al., 2015; Zong et al., 2017; Kim et al., 2018), polymerase chain reaction/restriction endonuclease (PCR/RE) (Feng et al., 2013; Lu and Zhu, 2017), annealing at critical temperature PCR (ACT-PCR) (Hilioti et al., 2016; Hua et al., 2017), high-resolution melting (HRM) (Thomas et al., 2014), quantitative real-time PCR (qPCR) (Peng et al., 2018), and droplet digital PCR (ddPCR) (Peng et al., 2020) are commonly used to detect gene-edited products. However, these detection methods have difficulty satisfying the requirements for sensitivity, rapidity, portability, low cost, and on-site detection at the same time. In view of these limitations, there is an urgent need for the method that can directly detect gene-edited products in the field without complex instruments.

The CRISPR/Cas12a system, a class 2 type V-A CRISPR**/**Cas system (Zetsche et al., 2015), performs *trans*-cleavage on nontargeted single-stranded DNA (ssDNA) upon the formation of the Cas12a/CRISPR RNA (crRNA)/target DNA ternary complex, determining whether there is a target based on the ssDNA (Chen et al., 2018; Li et al., 2018, 2019). Therefore, the Cas12a/crRNA system has been applied for nucleic acid detection (He et al., 2020; Lukas et al., 2020; Tao et al., 2020; Fan et al., 2021; Niu et al., 2021). For the detection of gene-edited products, Xiao et al. (2020) developed a CRISPR/Cas12a system to screen 23 CRISPR/Cas9-induced biallelic mutants in Thp-1 cells and detect mutants with 1–5 base insertions (synthesized commercially). However, commercially synthesized mutants are quite different from actual mutant materials. To date, there are few reports on the detection of single-base mutants in gene-edited products.

In this study, we developed a portable visualization method coupled with the CRISPR/Cas12a system, named Cas12aFVD (fast visual detection), to detect mutants in gene-edited rice, especially single-base mutants. In Cas12aFVD, the Cas12a/crRNA accurately and specifically binds with the target DNA to form a ternary complex, which activates the *trans*-cleavage of Cas12a to cleave the ssDNA fluorescent probe. The fluorescence signals can be monitored by the 7500 Real-Time PCR System (Thermo Fisher Scientific, USA) or the naked eye under blue light (470–490 nm). When Cas12aFVD is coupled with recombinase polymerase amplification (RPA), the fluorescent signals can be visualized in one tube within 40 min with high efficiency and sensitivity. This prevents contamination caused by uncapping and cross-sampling. In contrast to the traditional methods, this method can effectively resolve the problems faced in detection of gene-edited products. The developed Cas12aFVD biosensing platform provides a solution for rapid, visual, and on-site detection of gene-edited products.

## Materials and methods

### Materials and reagents

The gene-edited rice *CYP81A6* knockouts by using CRISPR/Cas9 and adenine base editor (ABE) were made in our laboratory. The NuClean Plant Genomic DNA Kit was purchased from CWBIOTECH Co., Ltd. (Jiangsu, China). The PCR reagent was purchased from Takara Biology Co., Ltd. (Dalian, China). The DNA Constant Temperature Rapid Amplification Kit (Basic) was purchased from Weifang Anpu Future Biotechnology Co., Ltd. (Weifang, China). The HiScribe T7 High Yield RNA Synthesis Kit and EnGen Lba Cas12a (Cpf1) were purchased from New England Biolabs (Ipswich, MA UK). RNA Clean & Concentrator-25 was purchased from Zymo Research (California, USA). All of the primers were synthesized by Sangon Biotech (Shanghai, China). The polyethylene filter membrane (aperture, 10 μm), silica gel adsorption membrane (aperture, 1 μm), and screw column were purchased from Hangzhou Laifeng Biotechnology Co., Ltd. (Hangzhou, China).

### Genomic DNA extraction and preparation of target DNA and crRNAs

The five mutants used in the present study were the homozygous mutants *Bel-1* (AG deletion), *Bel-2* (AGC deletion), *Bel-3* (A to G substitution), *Bel-4* (A insertion), and the heterozygous mutants *Bel-5* (A insertion). *Bel-1* and *Bel-2* were mutated at site one, *Bel-3* was mutated at site two, and *Bel-4* and *Bel-5* were mutated at site three. For each mutant, seed and leaf DNAs were extracted using the NuClean Plant Genomic DNA Kit and the rapid method, respectively. Rice ZH11 was used as a wild-type (WT) control.

**Figure 1:**
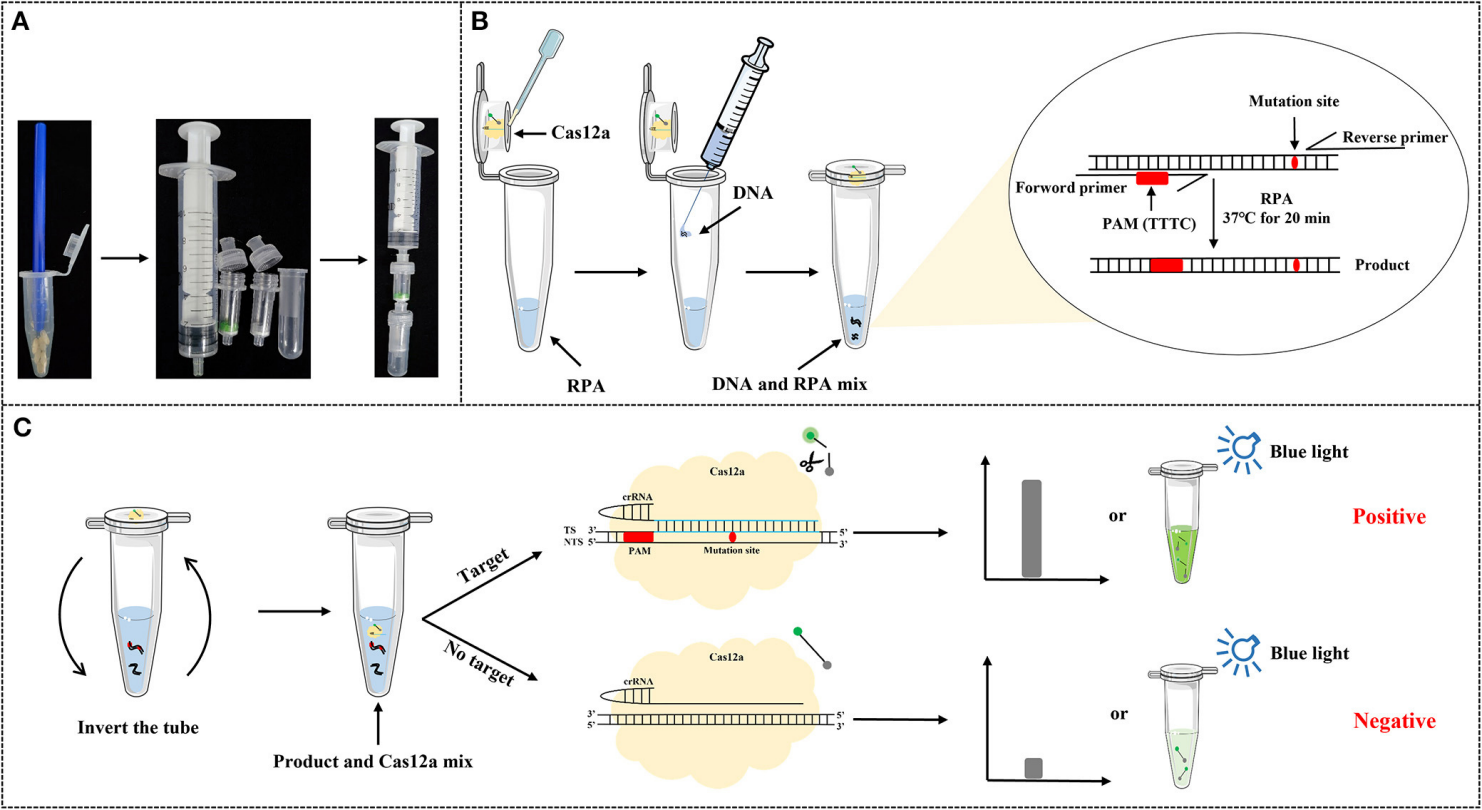
Schematic of the application of Cas12aFVD (fast visual detection) for the detection of gene-edited rice. **(A)** Rapid DNA extraction process. **(B)** Insertion of the protospacer adjacent motif (PAM) at an appropriate site through recombinase polymerase amplification (RPA). **(C)** After amplification, invert the tube to mix with Cas12a reagent for an additional 15 min. The fluorescence signals can be observed by the 7500 Real-Time PCR System (Thermo Fisher Scientific, USA) or by the naked eye under blue light (470-490 nm).

The rapid DNA extraction method (Zhang et al., 2013) was as follows. a) Fresh leaves (100 mg) were cut into pieces, and seeds (50 mg) were wrapped in tin foil and crushed with a hammer. Then, the samples were placed in a 1.5 ml centrifuge tube. Subsequent experiments were carried out using a portable device (Figure 1A). b) Then, 800 μl of extraction buffer (5 M guanidine thiocyanate, 50 mM Tris, 20 mM EDTA, 21.3 mM Triton X-100, 100 μg/ml RNase A; pH 6.4) was added, and the sample was ground for 1 min using a plastic rod. c) After homogenization for 30 s, the supernatant was poured into the filtration column, and the screw joint and the syringe were connected. The homogenized mixture was filtered by the air pressure generated by the syringe, then the filtrate was transferred to the adsorption column. The adsorption column was connected to the screw joint and the filtrate was further filtered by passing through the silica gel membrane, and gDNA was retained on the silica gel membrane. d) Then, 400 μl of buffer I (5 M guanidine thiocyanate, 50 mM Tris; pH 6.4) was added to the adsorption column, the syringe was pushed to filter the solution, and the filtrate was discarded. e) 200 μL of buffer II (10 mM Tris, 100 mM NaCl; pH 8.0) was added to the adsorption column, the syringe was pushed to filter the solution, and the filtrate was discarded. f) The adsorption column was moved to a new 1.5 ml centrifuge tube, 50 μl of water was added to the adsorption column, the syringe was pushed to filter the solution, and the filtrate was the extracted DNA sample. The entire extraction process took only 5 min.

For the five mutants, the crRNAs for guiding Cas12a were designed based on the 20 bp sequence information after the protospacer adjacent motif (PAM). The T7 promoter was contained in the synthetic double-stranded DNA (dsDNA) (Table 1). Then, after transcribing and purifying the RNA using RNA synthesis and Clean &Concentrator kits, the RNA samples were stored at −80°C.

**Table 1:** Primer, crRNA, and ssDNA probe sequences used in the experiments.

**Primer name**	**Sequence (5'-3')**
PCR1-F	GCCGGATCGTTTCGCCGT
PCR1-R	TCTCCATGAGCACGCTGAG
PCR2-F	AGATCATTTCCCGCACATCG
PCR2-R	ATGCTCTTCTTCTCGCCCTC
PCR3-F	GCAGGTCGTTTCGACGAGA
PCR2-R	Mutation site 2 and 3 shared the same reverse primer
RPA1-F	GCGCAACCTCCGCCGGATCGTTTCGCCGTG
RPA1-R	CCATGAGCACGCTGAGGGAGACCTCGAACA
RPA2-F	AGGTCGTCGACGAGATCATTTCCCGCACATCG
RPA2-R	CCGCTCCGCGTCGATCAGGCGGCGAAGGAA
RPA3-F	GGAGTTTAAGCAGGTCGTTTCGACGAGATC
RPA2-R	Mutation site 2 and 3 shared the same reverse primer
T7 primer	GAAATTAATACGACTCACTATAGGG
crRNA1	aauuucuacuguuguagauGCCGUGCCUGCUCUCCGCGCA
crRNA2	aauuucuacuguuguagauGCCGUGCUGCUCUCCGCGCAC
crRNA3	aauuucuacuguuguagauGACGAGAUCGUCCCGCACAUC
crRNA4	aauuucuacuguuguagauCCGCACAUCGAGCGCGGCCAA
ssDNA probe1	6-FAM-TTTTTT-TAMRA
ssDNA probe2	6-FAM-TTTTTT**-**BHQ1

### PCR assay

The primers used for PCR are listed in Table 1. Two thymine bases (TT) were inserted in the appropriate site of the forward primer, to introduce the Cas12a-recognized PAM (Sequences: TTTC). PCR was performed in a 20 μl reaction solution containing 4 μl of 5 × PrimeSTAR Buffer, 1.6 μl of dNTP mixture (10 mM), 0.4 μl each of forward and reverse primer (10 μM), 0.2 μl of DNA polymerase, 0.8 μl of DNA template, and 13 μl of ddH_2_O. The amplification protocol was as follows: 98°C for 5 min, 30 cycles of 98°C for 10 s, 60°C for 5 s, and 72°C for 15 s; and a final step of 72°C for 5 min. The amplified products were electrophoresed on a 1.5% agarose gel and cloned into the pJET1.2 vector (Thermo Fisher Scientific, USA), followed by DNA sequencing (Tsingke Biotechnology Co., Ltd.).

### RPA assay

The primers used for RPA are listed in Table 1. Double T was inserted in the appropriate site of the forward primer. Standard RPA was performed according to the instructions of the DNA Constant Temperature Rapid Amplification Kit (Basic). Each 20 μl reaction solution contained 11 μl of A buffer, one pellet, 0.8 μl (10 μM) each of forward and reverse primers, 1 μl of DNA template, 1 μl of B buffer, and 6.2 μl of ddH_2_O. The mixture was incubated in a water bath at 37°C for 20 min. The amplified products were extracted using phenol/chloroform/isoamylol and electrophoresed on a 1.5% agarose gel and cloned into the pJET1.2 vector, followed by DNA sequencing.

### CRISPR/Cas12a-based detection system

The CRISPR/Cas12a-based detection was performed in a 20 μl of reaction solution containing 50 nM Cas12a, 75 nM crRNA, 500 nM ssDNA probe 1 (ssDNA probe 2 for visual detection), 0.4 μl of ROX II, 2 μl of NEBuffer r2.1, 2 μl of DNA, and DNase/RNase-free water. The reaction was performed at 37°C for 60 min using the 7500 Real-Time PCR System (Thermo Fisher Scientific, USA), and fluorescence measurements were taken every minute.

### Cas12aFVD biosensing platform

In the visual detection experiment, there was no need to add ROX II. Cas12aFVD combined the RPA reaction with Cas12a cleavage in a portable device. Briefly, the RPA reagent was added to the bottom of the tube, and the Cas12a reagent was placed on the lid. To achieve all-in-one detection, 5, 10, 15, or 20 μl of RPA reagent was added. Water was added as a negative control (NC). The samples were processed at 37°C for 20 min by using a bottle heating insulation jacket (purchased from Foshan Niuwa Network Technology Co., Ltd.). After the RPA reaction, inverted the tube to mix with Cas12a reagent for target cleavage for an additional 15 min. The fluorescence could be observed under a blue light glue cutter (Monad, China) or a LUYOR-3415CV fluorescent protein excitation light source (LUYOR, USA).

## Results

### Schematic of the Cas12aFVD biosensing platform

As shown in Figure 1, after DNA extraction, a small amount of the DNA was added to a new tube that contained 20 μl of RPA reagent on the bottom and 20 μl of Cas12a reagent on the lid. After the RPA reaction, the tube was inverted to mix with Cas12a reagents. When the crRNA perfectly matched the target DNA, the Cas12a/crRNA/target DNA ternary complex was formed, which activates the *trans*-cleavage of Cas12a to cleave the ssDNA fluorescent probe. In the absence of mutants, crRNA fails to match the target DNA, which the *trans*-cleavage of Cas12a could not be activated, resulting in little or no fluorescence. We can distinguish positive and negative samples by the 7500 Real-Time PCR System or by the naked eye under blue light (470–490 nm). Thus, mutants could be distinguished from the control check (CK, wherein PAM was inserted) based on different fluorescence signals.

### PCR and RPA sequencing results

**Figure 2:**
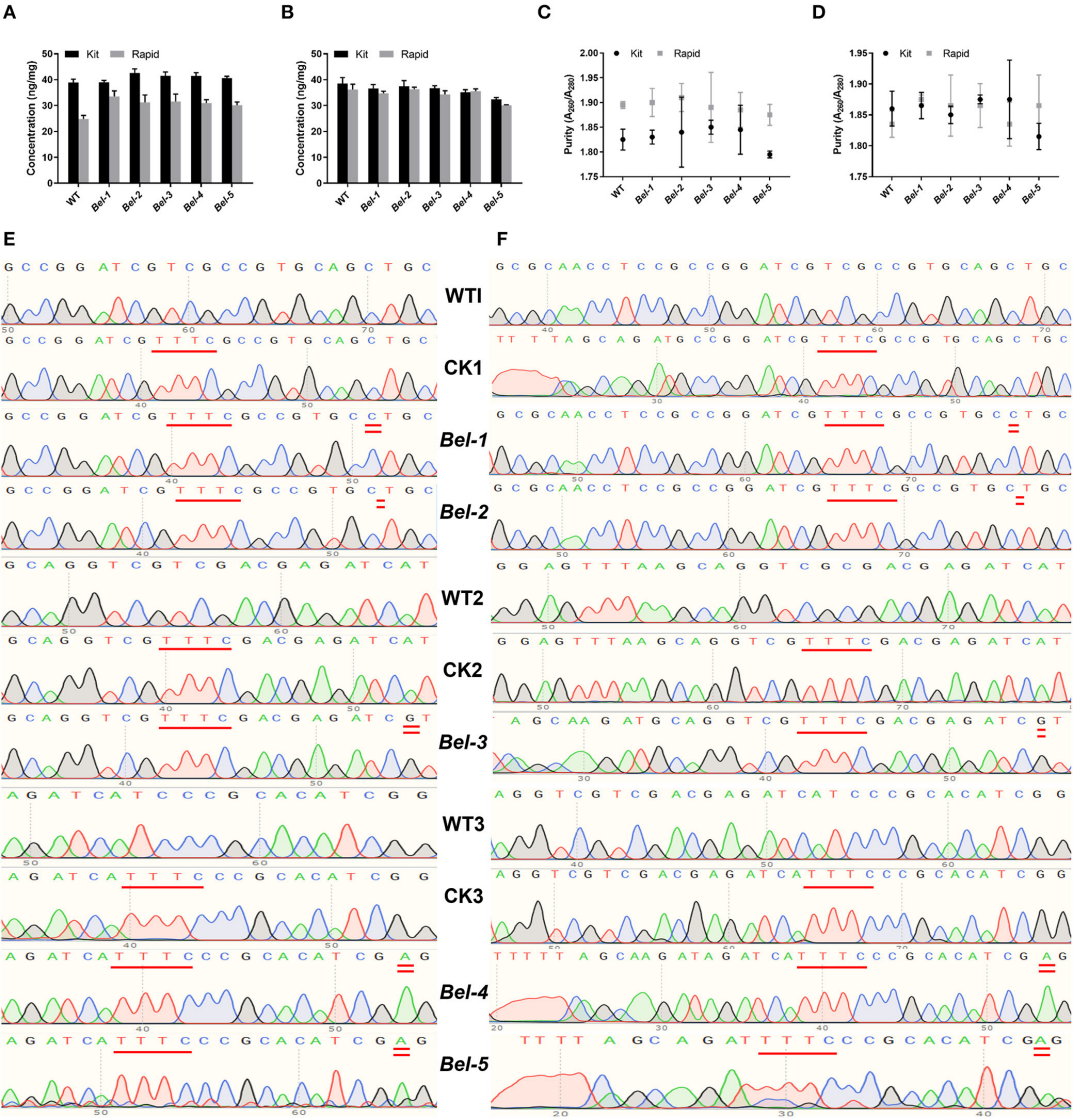
Concentration and purity of sample DNA extracted by two methods, and sequencing results for the polymerase chain reaction (PCR) and RPA products. **(A)** Seed DNA concentration. **(B)** Leaf DNA concentration. **(C)** Seed DNA purity. **(D)** Leaf DNA purity (*n* = 3, error bars show the mean ± SD). **(E)** PCR products of the wild-type (WT), control check (CK) (compared with the WT, a PAM was inserted in CK1, CK2, CK3), and five mutants. CK1, CK2, and CK3 represent three different editing sites. **(F)** RPA products of the WT, CK, and five mutants.

Kit and rapid methods were used to extract genomic DNA from 50 mg of seeds and 100 mg of leaves of WT and mutant rice. The concentration of the genomic DNA extracted by the rapid method was slightly lower than that of DNA extracted by the kit method (Figures 2A,B). However, the purity of the genomic DNA extracted by the rapid method was not as high as that of the DNA extracted by the kit method (Figures 2C,D).

Since there was no suitable PAM near the CRISPR/Cas9- and ABE-induced mutation site for Cas12a recognition, we needed to find an appropriate site to insert the PAM. To reduce the risk of introducing mutations, it was more appropriate to select the site with one T to form TTT. Inserting double T into the PCR and RPA forward primers, resulted in products containing PAM sites upon amplification (Li et al., 2018; Xiao et al., 2020). The sequencing results showed that the PAM was successfully inserted into the target sites (Figures 2E,F). WT1 and CK1, WT2 and CK2, and WT3 and CK3 represent three different edited sites. Compared with the WT, the PAM was inserted in CK1, CK2, and CK3.

### Optimization of the Cas12aFVD biosensing platform

Experiments were performed to verify the feasibility of detection and crRNA specificity, and to explore the optimal reaction concentration and time.

**Figure 3:**
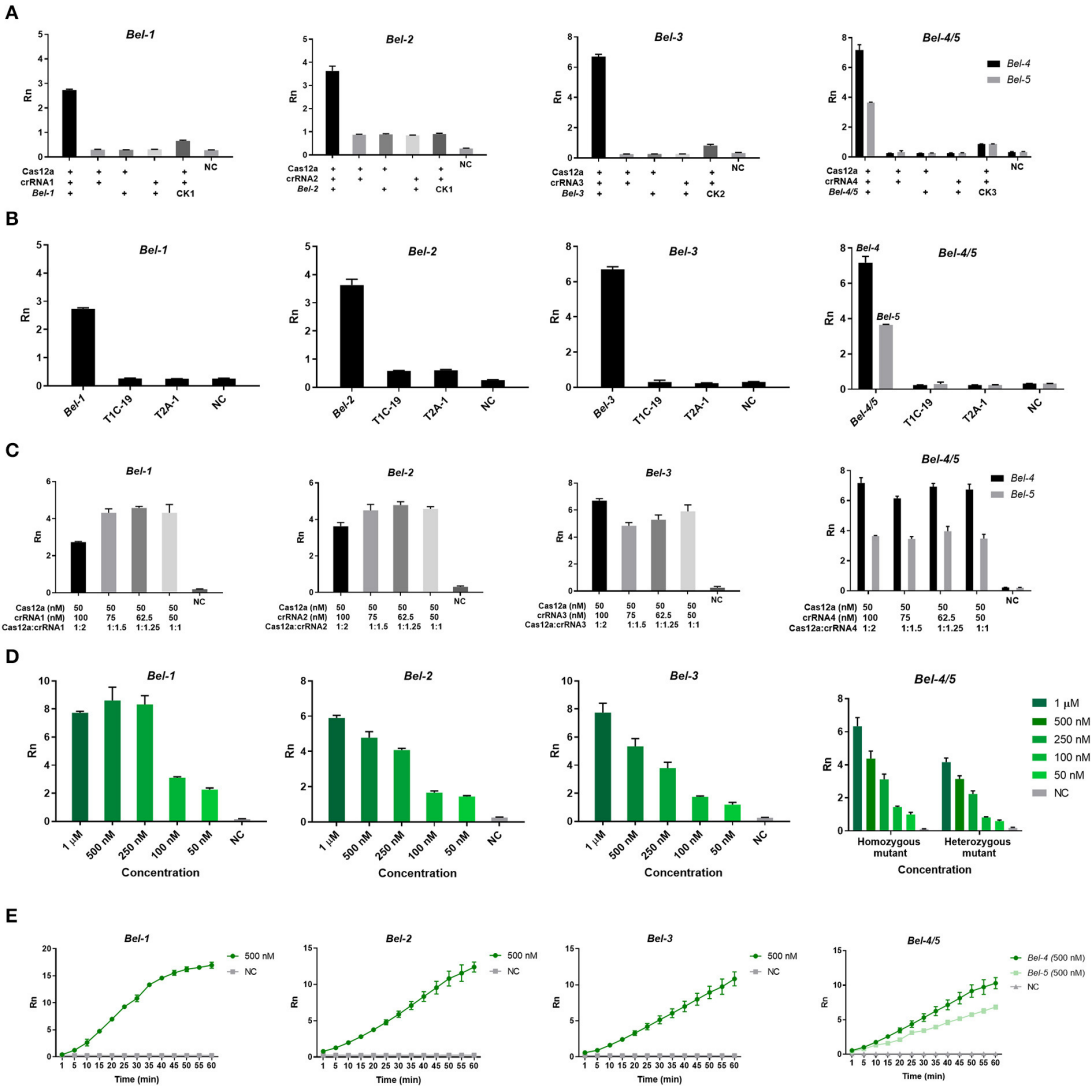
Optimization of the Cas12aFVD biosensing platform. Signals detected by the 7500 Real-Time PCR System. **(A)** Feasibility of the Cas12aFVD biosensing platform for nucleic acid detection. **(B)** Cas12a/CRISPR RNA (crRNA) detection specificity analysis. **(C)** Optimization of the molar ratio of Cas12a/crRNA. **(D)** Optimization of the concentration of ssDNA probe 1. **(E)** Cas12a/crRNA cleavage time. NC, negative control. *n* = 3, error bars show the mean ± SD.

**Figure 4:**
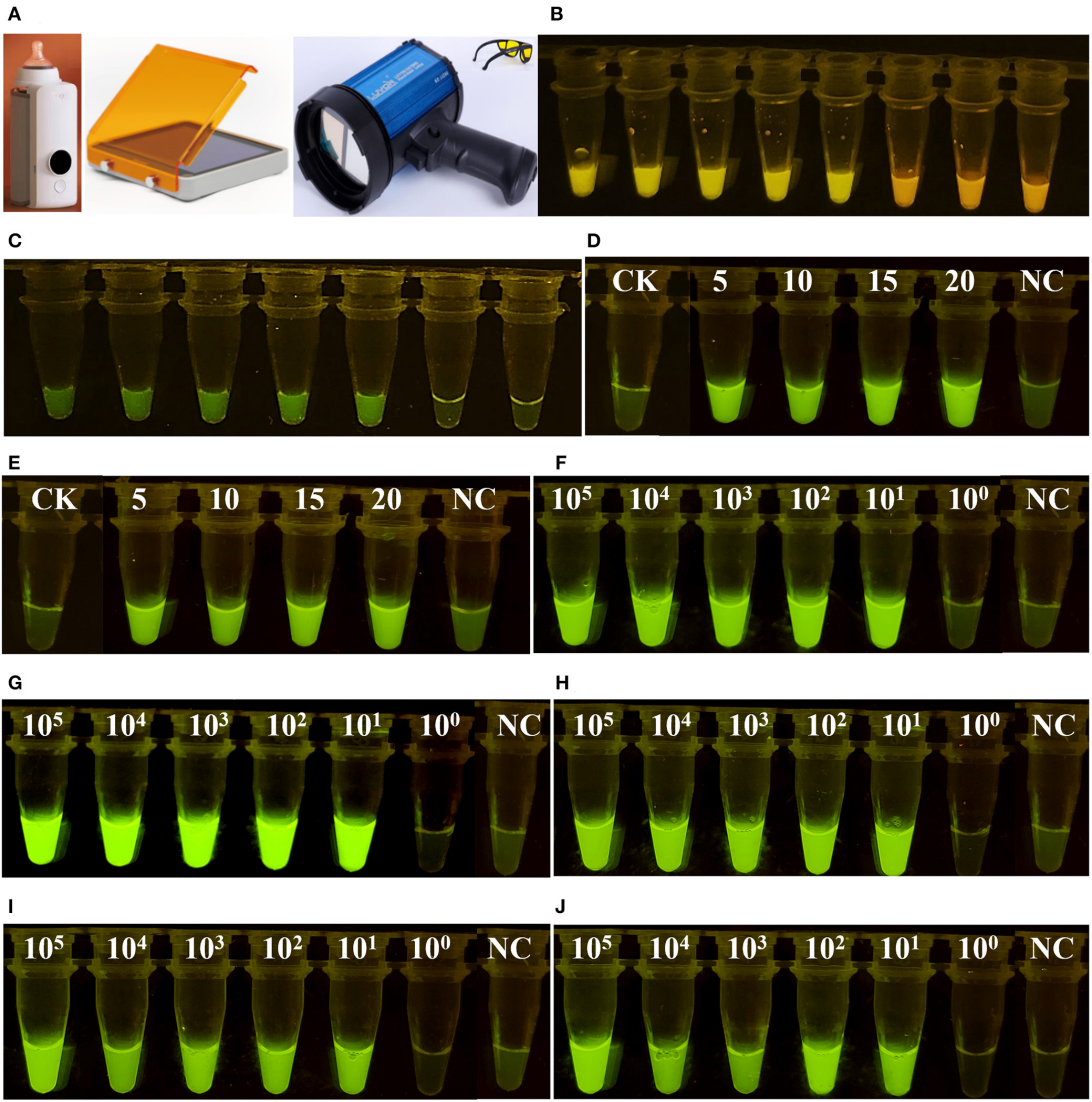
Visual detection of fluorescent signals. **(A)** Portable devices for heating and detecting fluorescence. **(B)** Visual detection of ssDNA probe 1. **(C)** Visual detection of ssDNA probe 2. **(D)** Optimization of the volume ratio of RPA and Cas12a. A total of 20 μl of Cas12a reagent combined with 5–20 μl of RPA reagent was employed. **(E)** The samples were processed for 12 h and then observed. **(F–J)** show six concentrations of *Bel-1, Bel-2, Bel-3, Bel-4*, and *Bel-5*, respectively.

As shown in Figure 3, only when Cas12a/crRNA bound specifically to the target DNA could the *trans*-cleavage of Cas12a be activated, based on the value of Rn (the ratio of the fluorescence of the reporter dye to that of the inert reference dye) (Figure 3A). This result suggests that the Cas12aFVD biosensing platform could be used for detection. Then, the specificity of Cas/crRNA was verified. *Bel-1*, the GM rice T1C-19, and the GM rice T2A-1 were added to the Cas12a/crRNA1 system to verify the cleavage specificity of Cas12a/crRNA1. The same experiments were performed for Cas12a/crRNA2, Cas12a/crRNA3, and Cas12a/crRNA4. *Bel-4* and *Bel-5* share the same crRNA. The results showed that cleavage could be performed only when the crRNA completely matched the target DNA (Figure 3B). Suitable concentrations of Cas12a, crRNA, and ssDNA probe 1 and an appropriate cleavage time for Cas12a were critical to improving detection sensitivity. Therefore, the fluorescence changes in Cas12aFVD with different concentrations of crRNA (50–100 nM) and ssDNA probe 1 (50 nM to 1 μM) and durations of 0–60 min were tested. There was no significant difference between 50 and 100 nM RNA, so 75 nM was chosen as the final concentration (Figure 3C), and 500 nM ssDNA probe 1 was chosen as the appropriate concentration (Figure 3D). For the cleavage time experiment, we found that the Rn of the mutants was more than ten times that of NC at 15 min (Figure 3E). Therefore, 15 min was selected as the detection time. However, when performing visual detection by portable devices (Figure 4A), the difference in fluorescence between the positive and negative colors for ssDNA probe 1 was difficult to distinguish. Therefore, we used ssDNA probe 2 instead (Figures 4B,C).

### Lower limit of detection of Cas12aFVD

**Figure 5:**
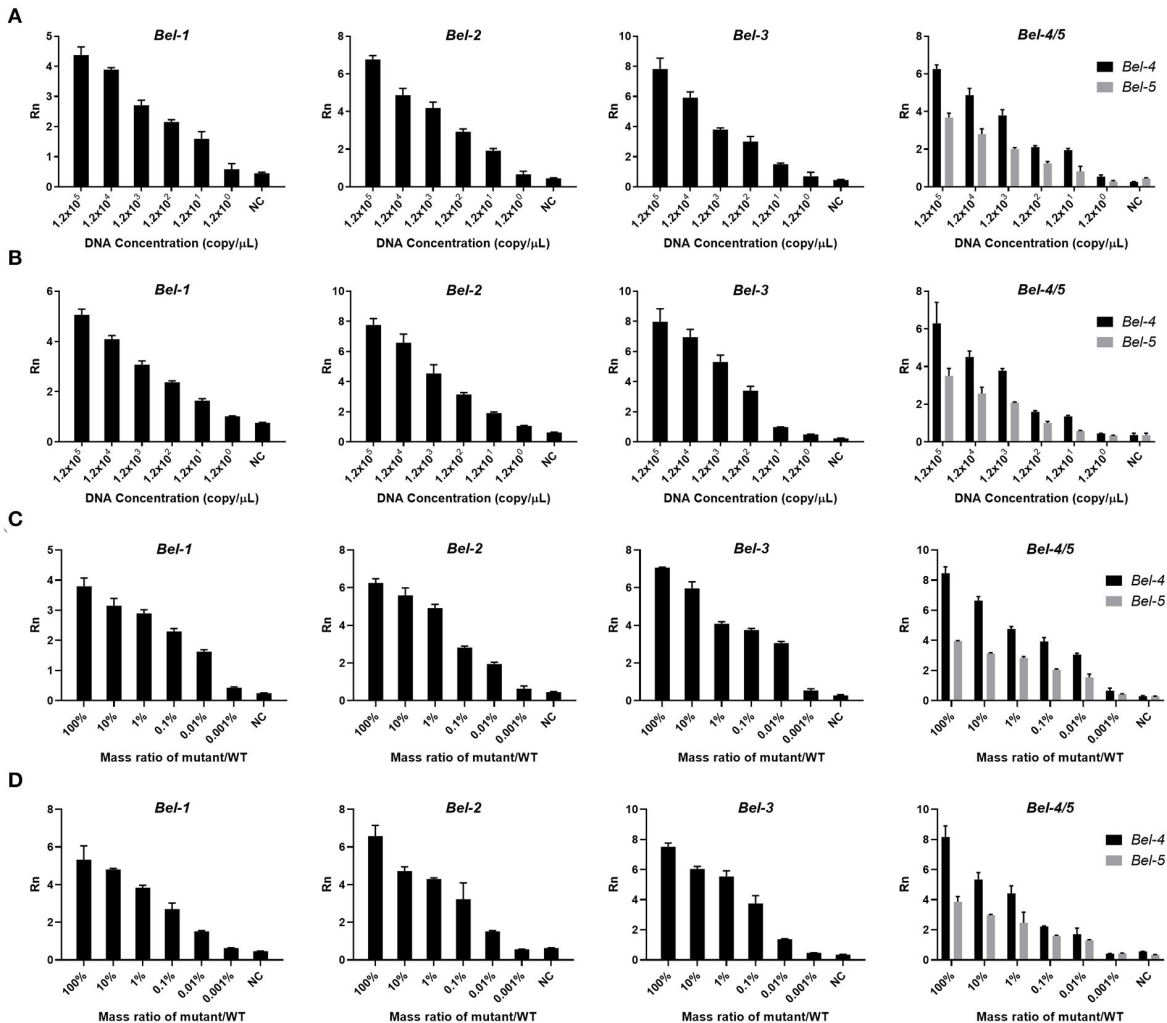
Lower limit of detection (LOD) of Cas12aFVD. **(A)** LOD of mutants amplified by PCR. **(B)** LOD of mutants amplified by RPA. **(C)** LOD of the mixture of mutant and WT amplified by PCR. **(D)** LOD of the mixture of mutant and WT amplified by RPA.

After determining the conditions, lower limit of detection (LOD) experiments were performed. Six concentrations of DNA template (10^5^, 10^4^, 10^3^, 10^2^, 10^1^, and 10^0^copies/μl) were used for the PCR and the RPA reaction. In addition, the mutants were mixed with the WT in a certain mass ratio (mass ratio of mutants and WT, 100, 10, 1, 0.1, 0.01, and 0.001%) for LOD determination. The LODs of Cas12aFVD were 12 copies/μl and 0.01% for the five mutants (Figures 5A–D).

### Visual detection

To make the detection process more convenient, portable devices were used for heating and detecting fluorescence (Figure 4A). The volume 5, 10, 15, and 20 μl RPA reagent all generated significant fluorescence (Figure 4D). To ensure the efficiency of the RPA reaction, 20 μl of RPA reagent was finally chosen. At some time points, the results were not observed. However, positive and negative samples could still be successfully distinguished after 12 h (Figure 4E). After determining the above conditions, we were able to detect the presence (bright green) or absence (light green) of the mutants by simply examining the color of the reaction solution by the naked eye (Figures 4F–J).

## Discussion

While collecting samples in the field, the instruments and laboratory conditions are generally not available for on-site detection. Taking samples back to the laboratory is inconvenient and time-consuming. Moreover, on-site detection is needed to obtain results as soon as possible. To avoid the need for bulky and complicated sensing instruments, portable instruments can be used to solve these problems.

First, a plant genomic DNA extraction kit and a centrifuge or vacuum pump are needed to extract DNA under laboratory conditions, but for on-site detection, we used the rapid method and portable device instead. This method made the DNA extraction process more convenient (Figure 1A), and the quality of the genomic DNA obtained was good enough (Figure 2). Therefore, the rapid method to extract DNA was suitable for the purpose. This method does not rely on bulky and expensive instruments and can meet the requirements for field sampling. Next, inserting double T into the PCR and RPA forward primers ensured that the amplified products contained a PAM site (Figure 2). The successful insertion of the PAM indicated that this approach enabled the Cas12aFVD biosensing platform to identify many mutant sites.

Then, in optimizing the detection conditions (Figure 3), we found that the formation of a ternary complex is a prerequisite for *trans*-cleavage. Coupled with the accurate recognition ability of Cas12a/crRNA for the target DNA, Cas12aFVD exhibited excellent specificity. Moreover, the signals of the heterozygous mutant were approximately half of those of the homozygous mutant. The mutants could be easily distinguished from the WT and NC, which also verified our hypothesis (Figure 3). Next, we used two methods to amplify the target. RPA showed good performance and sensitivity for amplification, which were comparable to those of PCR (Figure 5). In addition, RPA needs only one constant temperature and does not require strict temperature alternation. This makes the RPA reaction more convenient for on-site detection. It is generally thought that single-base mutants are difficult to detect. However, we were surprised to find that Cas12aFVD exhibited good detection performance for single-base mutants.

On-site detection contamination can easily occur when sampling and adding different reagents. We added two reagents into one tube to avoid contamination. Considering that the results may not be observed promptly, we processed the samples after 12 h and the mutants could be clearly distinguished from the WT and NC (Figures 4D,E). Considering the above experiments, the Cas12aFVD biosensing platform can be used in the detection of homozygous and heterozygous mutants and single-base mutants with high sensitivity and specificity.

In summary, we successfully developed “Cas12aFVD,” a portable rapid, visual, and portable platform for detecting mutants in gene-edited rice, especially single-base mutants, which can be used to perform on-site detection in one tube. Cas12aFVD was combined with the RPA method, enabling the fluorescent signals to be visualized in 40 min with an LOD of 12 copies/μl and 0.01%.

In recent years, CRISPR/Cas12a has been combined with rapid PCR, LAMP (Tao et al., 2020; Wu et al., 2020), electrochemical sensing (Wu et al., 2020; Fan et al., 2021; Li et al., 2021; Liu et al., 2021), lateral flow (Lu et al., 2020; Lukas et al., 2020), or aptamers (Niu et al., 2021; Qiao et al., 2021) to detect pathogens, proteins, small molecules, and so on. Chen et al. (2021) have developed a CRISPR/Cas12a-based biochip to detect single nucleotide polymorphisms (SNPs). However, for these methods, sophisticated electrochemical sensors need to be designed and used. Cas12aFVD has specificity and sensitivity, prevents contamination, and does not require complex sample handling, bulky instruments, tedious experimental steps, and professional analysis techniques, making it a promising and pioneering platform for the future detection of mutants in various GMOs, only by changing the crRNA. At the same time, Cas12aFVD can be combined with other techniques, such as colorimetry, electrochemistry, and immunology, providing technical support for laboratory and on-site detection. With further exploration, this CRISPR/Cas-based platform will have great application prospects.

## Data availability statement

The raw data supporting the conclusions of this article will be made available by the authors, without undue reservation.

## Author contributions

MW designed and performed the experiments and prepared the manuscript. XL provided the gene-edited rice. JY and ZW revised the manuscript. HW and XW conceived this project and supervised the experiments. All authors contributed to the article and approved the submitted version.

## Funding

This work was supported by the Central Public-interest Scientific Institution Basal Research Fund.

## Conflict of interest

The authors declare that the research was conducted in the absence of any commercial or financial relationships that could be construed as a potential conflict of interest.

## Publisher's note

All claims expressed in this article are solely those of the authors and do not necessarily represent those of their affiliated organizations, or those of the publisher, the editors and the reviewers. Any product that may be evaluated in this article, or claim that may be made by its manufacturer, is not guaranteed or endorsed by the publisher.
